# Case-based Workshop: Presentation of offenders with intellectual disabilities: towards an open and inclusive treatment

**DOI:** 10.1192/j.eurpsy.2024.106

**Published:** 2024-08-27

**Authors:** F. Saeedzadeh Sardahaee

**Affiliations:** ^1^Psychaitry, St Olav University Hospital; ^2^Psychiatric clinic, Lukasstiftelsen; ^3^Psychiatric clinic, Advansmedical, Trondheim, Norway

## Abstract

**Abstract:**

Legal frameworks and the challenges for treatment and rehabilitation of offenders with intellectual disabilities Speaker: Farzaneh Saeedzadeh Sardahaee, MD, PhD, Consultant psychiatrist St. Olav University Hospital, Trondheim, Norway

Lack of timely diagnostic and treatment for mild intellectual disability amongst offenders presents special challenges to clinicians and prison system alike. On the one hand, appropriate treatment for their psychiatric symptoms may not be implemented as they can be mislabeled primarily as behavioral issues. Different approaches to such perceived behavioral issues within judiciary (or prison) system and health care system is a potential conflict area. On the other hand, treatment and rehabilitation of offenders with intellectual disability require both resources and expertise that may not be readily available in prison systems, pre- or past sentencing. Furthermore, the scope of such challenges varies greatly based on different legal frameworks for sentencing offenders with intellectual disability within Europe.

In the first section an overview of Norwegian legal framework for offenders with intellectual disability is briefly presented. Then using the example of a young female offender with mild intellectual disability, drug dependence and multiple psychiatric morbidities, the speaker examines complexities of dual diagnoses, inter-disciplinary and multiagency cooperation follow-up and challenges faced in the recovery process. A brief introduction to the current Norwegian follow-up system for offenders with intellectual disability is discussed before examining recent changes in legal framework for sentencing offenders with intellectual disability in Norway, and its ramifications, as well as potential benefits for treatment and future rehabilitation of offenders. Finally, the speaker reflects on points for further improvement, especially considering the multi-agency nature of treatment and rehabilitation of offenders with intellectual disability.

**Disclosure of Interest:**

None Declared

